# Supplementary material for: Deep learning-based aberration compensation improves contrast and resolution in fluorescence microscopy
Source: Nat Commun. 2025 Jan 2;16:313. doi: 10.1038/s41467-024-55267-x (PMC11697233; doi:10.1038/s41467-024-55267-x)
Supplement: Supplementary file 2 — Description of Additional Supplementary Files [file 41467_2024_55267_MOESM2_ESM.pdf]

Supplementary Movie 1, Lateral views of synthetic phantom data restored by DeAbe vs. other methods. Phantoms consisting of randomly oriented and positioned dots, lines, spheres, spherical shells, and circles (ground truth, GT) were blurred to simulate microscopy data (Raw) and restored using blind deconvolution (Blind decon), Richardson-Lucy deconvolution with diffraction-limited PSF (RL Decon 1), Richardson-Lucy deconvolution with aberrated PSF (RL Decon 2), or our de-aberrating network (DeAbe). Lateral views though the volume are shown. Twenty iterations were used for RL deconvolution and 10 for blind deconvolution. See also **Fig. 1.**

Supplementary Movie 2, Axial views of synthetic phantom data restored by DeAbe vs. other methods. As in **Supplementary Movie 1**, but showing axial views through the volume.

Supplementary Movie 3, DeAbe restores images of *C. elegans* embryos expressing nuclear marker. Images were acquired with single view light sheet microscopy (iSPIM, 1.1NA). Left: raw data, middle: same data after 10 iterations of Richardson-Lucy deconvolution (RL Decon), right: restoration after DeAbe. Lateral views through the image volume are shown. See also **Fig. 3a**.

Supplementary Movie 4, DeAbe restores images of adult *C. elegans* expressing NeuroPAL;GCaMP6s. Images were acquired with spinning disk confocal microscopy. Left: raw data, right: restoration after DeAbe. Lateral views of individual color channels (1st – 3rd row) and combined channels (4th row) through the image volume are shown. Note contrast has been increased to better visualize dim nuclei, this results in a large background in the red channel in the last few planes. See also **Supplementary Fig. 18**.

Supplementary Movie 5, DeAbe restores images of NK-92 cells fixed and stained with Alexa Fluor 555 wheat germ agglutinin. Images were acquired with instant SIM. Volumetric maximum intensity projections (MIP), lateral views, and axial views through the image volume are shown in sequence. Left: raw data, middle: same data after restoration with DeAbe, right: same data after restoration with DeAbe+ (DeAbe followed by 20 iterations Richardson-Lucy deconvolution). See also **Fig. 3b-d**.

Supplementary Movie 6, DeAbe restores lateral views of live cardiac tissue expressing GFP-Tomm20. Images were acquired with two photon microscopy. Left: raw data, middle: after 20 iterations of Richardson-Lucy deconvolution (RL Decon), right: restoration after DeAbe. Lateral views through the volume are shown. See also **Fig. 3e-f**.

Supplementary Movie 7, DeAbe restores axial views of fixed tissue expressing tdTomato membrane marker. Images were acquired with two photon microscopy. Left: raw data, right: restoration after DeAbe.

Supplementary Movie 8, DeAbe ameliorates image degradation in mm-scale cleared mouse tissue embryo. Fixed and iDISCO-cleared E11.5-day mouse embryo was immunostained for blood vessels (CD31, magenta) and neurons (TuJ1, cyan). Rendering compares raw and DeAbe+ (attenuation compensated, DeAbe, and deconvolved) data. See also **Fig. 4a-d**.

Supplementary Movie 9, DeAbe enhances quantification of vessel orientation and alignment in mm-scale cleared mouse tissue embryo. Orientations (theta and phi) and 3D directional variance (DV) analysis on the blood vessel channel of the mouse embryo data in **Supplementary Movie 8**. Rendering compares raw and DeAbe+ (attenuation compensated, DeAbe, and deconvolved) results. See also **Fig. 4e-g**.

Supplementary Movie 10, Multi-step deep learning restores images of *C. elegans* embryos expressing nuclear (magenta) and membrane (green) markers. Images were acquired with single view light sheet microscopy (iSPIM, 1.1NA) and restored using a three-step deep learning pipeline. Maximum intensity projections (MIP, left column, shown only for nuclear channel) and single lateral plane 17.7μm into the volume (right column, both nuclei and membranes) are shown for raw (top row) and restored (bottom row) data. See also **Fig. 5a, b**.

Supplementary Movie 11, Multi-step deep learning improves image quality and cell segmentation for *C. elegans* embryos. The 80th time point volume (~320 min post fertilization) extracted from the time series data in **Supplementary Movie 10**. Top row shows raw image (top left) and restored image (top right) using three-step deep learning pipeline, with nuclei in magenta and membrane in green. Bottom row shows the automated cell segmentation based on the raw image (bottom left) and the automated cell segmentation followed by manual editing based on restored image (bottom right). 319 cells are automatically segmented in the raw data, but 421 cells are segmented based on manual editing of the multi-step deep learning result. Lateral views through the image volume are shown. See also **Fig. 5c**.

Supplementary Movie 12, Multi-step deep learning restores images of *C. elegans* embryos expressing ttx-3B-GFP, marking neurons and gut cells. Raw images (left) were acquired with single view light sheet microscopy (iSPIM, 0.8NA) and restored using three-step deep learning pipeline. Maximum intensity projections through time are shown; selected time points also show volumetric projections. See also **Fig. 5i**.

Supplementary Movie 13, Multi-step deep learning restores images of *C. elegans* embryos expressing nuclear marker. Images were acquired with single view light sheet microscopy (iSPIM, 1.1NA) and restored using three-step deep learning pipeline. From left to right were shown the raw images, Step 1 DeAbe, Step 2 Decon, and Step 3 Expan results. Lateral views through the volume are shown.

Supplementary Movie 14, Multi-step deep learning restores time-lapse images of *C. elegans* embryos expressing nuclear marker. Images were acquired with single view light sheet microscopy (iSPIM, 1.1 NA) and restored using three-step deep learning pipeline. From left to right were shown the raw images, Step 1 DeAbe, Step 2 Decon, and Step 3 Expan results. Maximum intensity projections through time are shown.

Supplementary Movie 15, DeAbe restores highly dynamic time-lapse images of live *C. elegans* expressing a GCaMP marker targeted to neurons. Images were acquired with instant SIM (1.15 NA) at 1.5 volumes/s over 1000 time points. Top: raw data; Bottom: restoration after DeAbe. Maximum intensity projections through time are shown.

Supplementary Movie 16, Higher magnification GCaMP data shown in Supplementary Movie 15. Images from the same dataset as **Supplementary Movie 15**, but higher magnification views of the nerve ring region from the period spanning 9.3 – 54.7 s, highlighting the rapid, fine details otherwise obscured in the raw images.

Supplementary Movie 17, Additional higher magnification GCaMP data shown in Supplementary Movie 15. Images from the same dataset as **Supplementary Movie 15**, but higher magnification views of the nerve ring region from another period spanning 423.5 – 493.6 s, highlighting the rapid, fine details otherwise obscured in the raw images.
