# Supplementary material for: Deep learning-based aberration compensation improves contrast and resolution in fluorescence microscopy
Source: Nat Commun. 2025 Jan 2;16:313. doi: 10.1038/s41467-024-55267-x (PMC11697233; doi:10.1038/s41467-024-55267-x)
Supplement: Supplementary file 20 — Reporting Summary [file 41467_2024_55267_MOESM20_ESM.pdf]

nature portfolio

Corresponding author(s): Min Guo

Last updated by author(s): Nov 2, 2024

Reporting Summary

Nature Portfolio wishes to improve the reproducibility of the work that we publish. This form provides structure for consistency and transparency in reporting. For further information on Nature Portfolio policies, see our Editorial Policies and the Editorial Policy Checklist.

Statistics

For all statistical analyses, confirm that the following items are present in the figure legend, table legend, main text, or Methods section.

n/a	Confirmed	
☐	☒	The exact sample size (*n*) for each experimental group/condition, given as a discrete number and unit of measurement
☐	☒	A statement on whether measurements were taken from distinct samples or whether the same sample was measured repeatedly
☐	☒	The statistical test(s) used AND whether they are one- or two-sided<br>*Only common tests should be described solely by name; describe more complex techniques in the Methods section.*
☒	☐	A description of all covariates tested
☒	☐	A description of any assumptions or corrections, such as tests of normality and adjustment for multiple comparisons
☐	☒	A full description of the statistical parameters including central tendency (e.g. means) or other basic estimates (e.g. regression coefficient) AND variation (e.g. standard deviation) or associated estimates of uncertainty (e.g. confidence intervals)
☐	☒	For null hypothesis testing, the test statistic (e.g. *F*, *t*, *r*) with confidence intervals, effect sizes, degrees of freedom and *P* value noted<br>*Give P values as exact values whenever suitable.*
☒	☐	For Bayesian analysis, information on the choice of priors and Markov chain Monte Carlo settings
☒	☐	For hierarchical and complex designs, identification of the appropriate level for tests and full reporting of outcomes
☒	☐	Estimates of effect sizes (e.g. Cohen's *d*, Pearson's *r*), indicating how they were calculated

Our web collection on statistics for biologists contains articles on many of the points above.

Software and code

Policy information about availability of computer code

Data collection	The simulated data of phantom objects and aberrated images were generated with MATLAB R2022b. The code is publicly available at (https://github.com/eguomin/DeAbePlus). The synthetic images (SI Fig. 13a) were generated with MATLAB 2019b.<br>Experimental images were collected with the home-built light-sheet microscopes (diSPIM and/or iSPIM, and AO-LLSM) and commercial confocal microscope (Zeiss LSM 880), spinning-disk confocal microscope (Nikon, Ti-e), two-photon microscope (Leica SP8) and instant structured illumination microscope system (VisiTech Intl, Sunderland, UK) as described in the Methods section of our paper.
Data analysis	3D-RCAN and CARE software was installed from GitHub (https://github.com/AiviaCommunity/3D-RCAN; https://github.com/CSBDeep/CSBDeep). No version numbers are defined for CARE or RCAN. We used Python version 3.7.0 for all neural networks. Image deconvolutuion and multiview fusion were performed with diSPIMFusion (https://github.com/eguomin/diSPIMFusion). Richardson-Lucy (RL) deconvolution was performed within MATLAB R2022b using our previously developed deconvolution package (https://github.com/eguom/regDeconProject). Blind deconvolution was performed within MATLAB R2022b using MATLAB function deconvblind with default settings (https://www.mathworks.com/help/images/ref/deconvblind.html). Mask-RCNN (https://github.com/matterport/Mask_RCNN) was used for nuclear segmentation, and seeded watershed algorithm (https://github.com/danielsnider/Simple-Matlab-Watershed-Cell-Segmentation) was used for membrane segmentation. Manual editing on the membrane segmentation was performed within the ImageJ plugin Labkit (https://imagej.net/plugins/labkit/). Image Decorrelation analysis was perform using the code from GitHub: https://github.com/Ades91/ImDecorr. Other image analysis (including decay correction, vessel orientation analysis, SSIM, PSNR, DCTS, RMSC calculations, etc) were performed within MATLAB R2022b.

For manuscripts utilizing custom algorithms or software that are central to the research but not yet described in published literature, software must be made available to editors and reviewers. We strongly encourage code deposition in a community repository (e.g. GitHub). See the Nature Portfolio guidelines for submitting code & software for further information.

Data

Policy information about availability of data

All manuscripts must include a data availability statement. This statement should provide the following information, where applicable:

- Accession codes, unique identifiers, or web links for publicly available datasets
- A description of any restrictions on data availability
- For clinical datasets or third party data, please ensure that the statement adheres to our policy

The data that support the findings of this study are included in Supplementary Figs. 1–39 and Supplementary Videos 1–17. Some representative data from the figures (Fig. 2a, Supplementary Figs. 16, 30) are publicly available at https://zenodo.org/record/8424246. Other datasets (training data and intermediate data for deep learning) are available from the corresponding author upon reasonable request due to their large file size.

Human research participants

Policy information about studies involving human research participants and Sex and Gender in Research.

Reporting on sex and gender	N/A
Population characteristics	N/A
Recruitment	N/A
Ethics oversight	N/A

Note that full information on the approval of the study protocol must also be provided in the manuscript.

Field-specific reporting

Please select the one below that is the best fit for your research. If you are not sure, read the appropriate sections before making your selection.

- [x] Life sciences
- [ ] Behavioural & social sciences
- [ ] Ecological, evolutionary & environmental sciences

For a reference copy of the document with all sections, see nature.com/documents/nr-reporting-summary-flat.pdf

Life sciences study design

All studies must disclose on these points even when the disclosure is negative.

Sample size	For SSIM and PSNR quantification (Fig. 1 and S. Figs 1-9), statistical results (Means and standard deviations) were obtained from N= 100 independent simulations. For decorrelation analysis, statistical results (Means and standard deviations) were obtained from N= 12 (Fig. 2f), N=15 (Fig. 2m), and N=3 (S. Fig. 13f) experiments. For cell segmentation and counting analysis, statistical results (Means and standard deviations) were obtained from N= 3 different embryos (Fig. 5d and S. Fig. 31).
Data exclusions	No data were excluded from the analysis.
Replication	The reproducibility of the experimental findings was verified by imaging distinct fixed and live samples with sample size N>=3. Time-lapse imaging experiments were repeated at least 3 times, with similar results obtained each time. All attempts at replication were successful.
Randomization	In this study, samples were not allocated into different experimental groups.
Blinding	The investigators were not blinded to group allocation during data collection and data analysis. We don't think blinding is relevant in this study and we demonstrated the technique on distinct samples by collaborating with different research groups.

Reporting for specific materials, systems and methods

We require information from authors about some types of materials, experimental systems and methods used in many studies. Here, indicate whether each material, system or method listed is relevant to your study. If you are not sure if a list item applies to your research, read the appropriate section before selecting a response.

Materials & experimental systems

n/a	Involved in the study
	☒ Antibodies
	☒ Eukaryotic cell lines
☒	Palaeontology and archaeology
	☒ Animals and other organisms
☒	Clinical data
☒	Dual use research of concern

Methods

n/a	Involved in the study
☒	ChIP-seq
☒	Flow cytometry
☒	MRI-based neuroimaging

Antibodies

Antibodies used	The mouse embryos were stained with monoclonal antibody against PECAM1 (CD31, clone MEC 13.3, Cat# 553700, BD Pharmingen, 1:200 dilution) and monoclonal anti-β-tubulin III (TuJ1)) antibody (clone 2G10, Cat# T8578, Sigma-Aldrich, 1:500 dilution) in blocking buffer overnight. After washing with 0.2% Triton/PBS, the embryos were stained with secondary antibodies with Alexa 488 goat anti-rat IgG and Alexa 594 goat-anti-mouse IgG (1:250, Invitrogen, Carlsbad, CA) in blocking buffer overnight.
Validation	The antibodies have been validated from the reference below<br>1) Monoclonal antibody against PECAM1 (BD Pharmingen, Cat# 553700)<br>(a) Vendor website: https://www.bdbiosciences.com/en-us/products/reagents/flow-cytometry-reagents/research-reagents/single-color-antibodies-ruo/purified-rat-anti-mouse-cd31.550274<br>(b) Vendor statement: The MEC13.3 antibody specifically recognizes CD31, also known as PECAM-1 (Platelet Endothelial Cell Adhesion Molecule-1).<br>(c) Reference: Norika Liu, Naofumi Kawahira, et al., Notch and retinoic acid signals regulate macrophage formation from endocardium downstream of Nkx2-5. Nature Communications (2023).<br>2) monoclonal anti-β-tubulin III (TuJ1)) antibody (Sigma-Aldrich, Cat# T8578)<br>(a) Vendor website: https://www.sigmaaldrich.cn/CN/en/product/sigma/t8578<br>(b) Vendor statement: Tubulin β 3 class III (TUBB3) also known as β-Tubulin III, is encoded by the gene mapped to human chromosome 16q24.3. TUBB3 protein expression is restricted to neurons. Monoclonal Anti-β-Tubulin III (neuronal) antibody produced in mouse has been used in Immunofluorescence and Immunocytochemistry.<br>(c) Reference: N Bali, J M Arimoto, et al., Progesterone antagonism of neurite outgrowth depends on microglial activation via Pgrmc1/S2R. Endocrinology (2013)

Eukaryotic cell lines

Policy information about cell lines and Sex and Gender in Research

Cell line source(s)	NK-92 cells (ATCC®, CRL-2407™) and PtK2 cells (ATCC®, CCL-56™) were used in this study.
Authentication	None of the cell lines used were authenticated.
Mycoplasma contamination	The cell lines were not tested for mycoplasma contamination.
Commonly misidentified lines (See ICLAC register)	No commonly misidentified cell lines were used in this study.

Animals and other research organisms

Policy information about studies involving animals; ARRIVE guidelines recommended for reporting animal research, and Sex and Gender in Research

Laboratory animals	Seven nematode strains (BV514, OD58, DCR6268, SLS164, OH15500, ABA0001, and ZIM1997) were used in this study. All worms were kept at 20°C, and grown on NGM media plates seeded with E. coli OP50. For strains BV514, OD58, DCR6268, and SLS164, embryos were dissected from gravid adults for imaging. For strain OH15500 , ABA0001, and ZIM1997, young adult worms (with 2 or less visible eggs in their uterus) were picked and immobilized or fixed for imaging. For AO benchmark, zebrafish embryos (5 dpf) were fixed and used for imaging. Mouse embryos (E11.5 day) were used for cleared imaging and Mouse tissues (fixed mouse liver samples and fresh ex-vivo mouse heart muscle strips) were used for two-photon microscopy imaging.
Wild animals	This study didn't involve wild animals.
Reporting on sex	Information had not been collected and this study didn't involve sex-based analysis.
Field-collected samples	This study didn't involve samples collected from the field.
Ethics oversight	All animal studies were performed in a manner consistent with the recommendations established by the Guide for the Care and Use of Laboratory Animals (National Institutes of Health), and all animal protocols were approved by the Animal Care and Use Committees in NCI or NHLBI.

Note that full information on the approval of the study protocol must also be provided in the manuscript.
